# Buffalo Medical College, and New York Annalist

**Published:** 1849-06

**Authors:** 


*Buffalo Medical College, and New York Annalist.*—The Editor of the Annalist in his comments, May 1st, on an article contained in the editorial department of this Journal in the No. for April, inadvertently does the latter, as it appears to us, injustice. Our respected contemporary quotes our remark that "justice to the interests of the College, seems to require this policy, (*i. e* return to the four months' term,) so long as other, and especially proximate institutions, continue to adhere to a four months' term," and upon this statement bases an argument, that the interests of the institution are at variance with the interests of medical education.

The injustice to which we allude, consists in this, viz., that regard for the *interests* of the college is made to appear as the sole reason for our advocacy of the change. This was assigned as a reason, it is true, but in the next paragraph we go on to remark that "after testing the practical working of the plan of continuing lectures for a longer period, we are less disposed, than heretofore, to urge its importance." The reasons for this modification of our views are then given, which, as it seems to us, it would have been but fair in the Editor of the Annalist to have quoted, while he does us the honor to quote tne portion of our article which appears to suit the purpose of his argument. These reasons, as our readers will perceive by reference to the article referred to, have no connection with the interests of the college, but relate entirely to the interests of the medical student, and of medical education.

Having said thus much, as due to ourselves, we copy the portion of the article by our brother of the Annalist, containing his reasoning on the passage quoted from our article, and will then offer a few remarks upon the soundness of his ratiocination:—

But if medical colleges were restricted entirely to the business of *teaching*, wi:hout any formal admission of the candidates into the ranks of the profession, does our respected cotemporary believe that short terms would constitute any special recommendation in the mind of the student? If the diploma was entirely out of view, leaving the student the simple proposition, that for $60, more or less, he can have five months instruction at Buffalo, and onlyfour at Geneva or Cleveland, does any one suppose that the privilege of taking an additional month at the same price would constitute an objection? But it is a universal rule, that the mass of mankind, wanting any given thing, will obtain it where it can be had the cheapest. And so long as it is the ultimate object of the student to obtain his diploma, and it can be had at one school at the end of a four month's course, while at another five or six months is required, the former will have a strong and obvious advantage over the latter. Thus, the very fact, that *justice* to the *interests* of the Buffalo College requires a return to the old four month's course, is clear and obvious proof that the connection of licensing powers with institutions for medical teaching, is wrong, and practically subversive of the best interests of the profession. Although we have constantly advocated a longer Lecture Term in our Medical Colleges, yet that measure by itself, unaccompanied by others, has ever seemed too much like adding *a new piece to an old garment.* The truth is, that medical colleges, like all other schools, should be simply *teaching* institutions, divested of every extraneous power or privilege; so that their competition, instead of being to see which can afford the most attractive facilities for conferring Degrees, shall be a struggle to see which shall present the most extended and complete course of medical instruction for a given amount of money.

The argument of the writer hangs upon the postulate, that the chief object with the medical student in attending a medical school, is to secure a diploma, irrespective of the character or amount of the instruction he expects to receive. Assuming this, and applying the common maxim that a man will purchase what he wants where he can get it the cheapest, he arrives at the deduction that those schools will be best patronised where the honors are bestowed upon the easiest terms. Hence, the *interests* of schools being dependent on the patronage they receive, the struggle of competition is not to "be which shall present the most extended and complete course of medical instruction for a given amount of money," but, "which can afford the most attractive facilities for conferring degrees." *Ergo*—the "connection of licensing powers with institutions for medical teaching is wrong, and practically subversive of the best interests of the Profession."

This, if we understand the scope of the argument, is the chain of reasoning which a fair analysis of the passage above quoted, develops.

Now, in the first place, we think the first postulate involves an imputation on the character of medical students, as a body, which cannot be sustained. The imputation extends, also, to the body of the medical profession, since they all were, whilom, medical students. Is it in accordance with the reminiscences and observations of the majority of our readers, that candidates for admission into the ranks of the profession, are actuated in their preparatory studies by no higher or better motives than to obtain a license to practise? We trow not. That this cannot be the case is proved by various facts which militate directly against the position upon which the writer rests his argument. If the writer's reasoning were correct, would it be true that institutions which, from their situations, and other supposed advantages, as respects means of instruction, command the largest amount of patronage, in other words, the largest number of pupils? Why is it that students aunually assemble in such large numbers at the Pennsylvania and Jefferson schools? Is it because they are able to get Degrees at these Institutions with greater facility than at others? Moreover, both these Institutions have extended their term of lectures: how does this comport with the assertion of which we are speaking! We can refer the writer to an Institution that requires two courses of only three months, and yet there are few, if any Institutions in the United States which do not have larger classes than this. How is this to be accounted for, on the writer's supposition!

Again, if it be indeed true, as the writer implies, that the point of competition among medical schools is, which shall afford the most attractive facilities for granting diplomas, why is it that the fact is not made to appear in their annual circulars? Assuredly if this be true, and the great object is to receive patronage upon this ground, it would not be concealed —there would be no motive for concealment, but every motive for every institution to have its advantages in this respect, well, and widely known. This must be conceded; now we put the question, where is the institution that gives any intimation that it will graduate with more facility than others! We could multiply considerations of a like pertinency, but those which have been mentioned suffice, as we think, with all respect, for a *reductio ad absurdam*. So far from medical students being actuated by the unworthy aims imputed to them, we believe the majority earnestly desire to qualify themselves for their duties, to the full extent of their resources. The facts which have been adduced, as we think, demonstrate this to be the case. They will flock in greatest numbers to the institutions where they suppose the opportunities for acquiring knowledge are most abundant. We do not say there are no exceptions to this rule. We contend only for the rule. Many medical students pursue their studies under embarrassments and obstacles, especially those incident to the *res angusta domi*. The necessity for economy may stand in the way of preferring a five or six, to a four months term, many feeling compelled to choose to labor with additional assiduity during the latter period, and save the expense of one or two additional months absence from home; for, it is to be recollected, (which the writer appears to overlook) that the cost of a lengthened term is necessarily increased, notwithstanding the teachers' fees remain the same. Other circumstances may also render a four months' term desirable with many; but they who only desire to graduate, and are careless of availing themselves of educational advantages, are not the class who would be inconvenienced by, or opposed to a longer period of lectures. They would generally, indeed, give the preference to a protracted session, especially if in the city, because most of this class are given to dissipation, or, what is called, pleasure.

Collegiate medical degrees are desired by most candidates for the medical profession; but why are they desired? Chiefly because they are considered honorable insignia, not because they confer a license to practice. Attendance at a single course of lectures is considered desirable by many who are unable, from straightened means, or other causes, from attending two courses, and becoming candidates for graduation. Why should this be so, if not for the actual advantages enjoyed, and the credit which even attendance upon a course of lectures, without a degree, confers! If the license were the exclusive purpose, why not seek it from state and county censors, who are not generally thought to be more rigid in their requirements than a board of Professors! Indeed, a license in many, if not most of the states of the Union, is superfluous, owing to the fact that legislation imposes no restrictions upon practicing medicine without it.

Without pursuing the subject farther at this time, we embrace this opportunity to say a few words respecting the tenor of remarks occasionally made upon the character of medical colleges.

We have no disposition to charge upon the able Editor of the Annalist any lack of courtesy, but the article which has given occasion for these comments appears to partake somewhat of a tenor of invective, by no means peculiar to the writer. The *interests* of a medical school are spoken of, as if this term applied wholly to the pecuniary emoluments of the teacher's office. Teachers are often indirectly, if not directly, accused of being ready to sacrifice the interests of medical education to the lust for lucre—of being influenced solely by mercenary considerations in the dispensation of the honors and privileges of the institutions under their charge. These are not trifling accusations, and should not, in common charity, be flippantly uttered. It would be difficult to assign a valid reason why the labors of a medical teacher should not command pecuniary compensation, as well as for ordinary professional services; and, yet, poorly as the latter are provided for, the former are still more inadequately recompensed. Medical teaching, surely, is far from offering a tempting field for avarice. A little observation suffices to show that the privilege of endeavoring to be useful in that sphere, oftener requires sacrifices, than confers gain. The same amount of time and effort in practice would be vastly more lucrative. But, be this as it may, or were it quite otherwise, we protest against attacks upon the motives and aims of those engaged in this line of duty, which would be considered neither decorous nor admissible, when applied to the same individuals, in the capacity of practitioners. The rules of medical ethics are violated in the former case, equally as in the latter. We beg to commend this as a topic of reflection not always sufficiently considered.

But, it may be said, if corruption and malversations do exist, they deserve to be stigmatised. Granted. We shall not argue for the propriety of withholding merited censure in any case. But it may be claimed, in behalf of medical schools, in common justice, that attacks be coupled with charges and specifications. Instead of wholesale vituperation, let it be distinctly stated what institutions are guilty of abuses, and what the abuses are. Let, not only the profession at large, but those institutions which are not obnoxious to censure, have the benefit of the facts upon which insinuations, referring alike to all schools, are based. It may then be ascertained how much of the opprobium heaped on medical colleges is deserved, and the offending institutions being designated, it will be known where confidence should be bestowed, or withheld, and where reforms are needed.

On this subject, a friend had prepared some resolutions to submit at the last meeting of the American Association, which, owing to the amount of other miscellaneous business, he concluded not to present. As these resolutions express views which appear to us important and just, and in which medical schools, and the profession generally, are alike interested, we have requested them for publication. We commend them to the consideration of our readers, and at the next meeting of the Association we hope they will be submitted and acted upon.*

* The publication of the resolutions referred to is reserved for the next Number.

